# Erzhi Tiangui formula ameliorates ovarian aging by enhancing antioxidant defense via Nrf2/HO-1 signaling

**DOI:** 10.1186/s13048-026-02202-x

**Published:** 2026-07-18

**Authors:** Xiufang Li, Zhongqing Wang, Xiuqing Zhang, Huidan Wang, Xu Han, Ye Zheng, Mei Sun, Haicui Wu, Fang Lian

**Affiliations:** 1 State Key Laboratory of Reproductive Medicine and Offspring Health, Center for Reproductive Medicine, Institute of Women, Children and Reproductive Health, Shandong University, Jinan, China; 2 The First Clinical College, Shandong University of Traditional Chinese Medicine, Jinan, China; 3 Jinan High-tech East District Hospital, Jinan, China; 4 Integrative Medicine Research Centre of Reproduction and Heredity, The Affiliated Hospital of Shandong University of Traditional Chinese Medicine, Jinan, China

**Keywords:** Erzhi Tiangui formula, granulosa cells, oxidative stress, Nrf2/HO-1 pathway, apoptosis, mitochondrial function

## Abstract

**Objective:**

This study aimed to investigate the mechanisms through which Erzhi Tiangui Formula (EZTG) mitigates age-related ovarian aging in mice through the Nrf2/HO-1 antioxidant pathway.

**Methods:**

A total of 100 C57BL/6J mice were divided into young control (YC, 6–8 weeks, *n* = 20) and aged groups (8–9 months, *n* = 80). Aged mice were randomized into four subgroups: control, low-(0.05 g/mL), medium- (0.2 g/mL), and high-dose (0.8 g/mL) EZTG groups (*n* = 20/group). After 10 days of treatment, ovarian morphology and follicular counts were assessed. On day 11, the remaining mice received an intraperitoneal injection of human menopausal gonadotropin (hMG, 15 IU), followed by human chorionic gonadotropin (hCG, 12 IU) 48 h later. 14 h after hCG administration, the mice were euthanized under deep anesthesia via cervical dislocation, and granulosa cells were collected. The following parameters were evaluated: oxidative stress markers (ROS), Nrf2/HO-1 pathway components (Nrf2, HO-1, Keap1), apoptosis factors (Bax, Bcl-2, cleaved Caspase-3), antioxidant enzymes (SOD, CAT, GPX1), and mitochondrial function (mitochondrial membrane potential, MMP; ATP). In vitro, KGN human granulosa cells were subjected to H₂O₂-induced oxidative stress with or without EZTG-containing serum, and Nrf2 was knocked down by siRNA to validate pathway specificity.

**Results:**

EZTG treatment significantly improved multiple parameters in a dose-dependent manner. Treated mice exhibited restored estrous cyclicity and enhanced follicular development with reduced atresia. At the cellular level, EZTG effectively decreased ROS accumulation while upregulating antioxidant enzymes, including SOD, CAT, and GPX1. Molecular analyses revealed activation of the Nrf2/HO-1 signaling pathway, as evidenced by increased Nrf2 and HO-1 expression alongside Keap1 downregulation. Additionally, EZTG exerted an anti-apoptotic effect, demonstrated by upregulated Bcl-2 expression and downregulated Bax and cleaved Caspase-3. Mitochondrial function assays further revealed that EZTG significantly improved MMP and ATP production in granulosa cells. Notably, the medium- and high-dose groups consistently outperformed the low-dose group across all measured outcomes, reinforcing a clear dose-response relationship.

**Conclusion:**

EZTG attenuates ovarian aging by coordinated activation of the Nrf2/HO-1 pathway, enhancing antioxidant defenses, inhibiting apoptosis, and restoring mitochondrial function in a dose-dependent manner. These findings provide mechanistic insights into EZTG’s potential as a therapeutic intervention for age-related ovarian decline.

**Clinical trial number:**

not applicable.

**Supplementary Information:**

The online version contains supplementary material available at https://doi.org/10.1186/s13048-026-02202-x.

## Introduction

Age is a crucial determinant of ovarian reserve and an independent factor in assessing female fertility. Current guidelines classify women over 35 as being of advanced maternal age [1], a group characterized by significantly reduced reproductive potential. With the trend toward delayed childbearing and increased life expectancy, the proportion of women in this category has risen sharply worldwide [2]. Although the mechanisms underlying age-related ovarian dysfunction are not yet fully understood, oxidative stress is widely recognized as a major contributor, playing a critical role in abnormal follicular atresia and declining embryo quality [3]. Aging is associated with elevated levels of reactive oxygen species (ROS) and a decline in antioxidant defense enzymes, which together impair ovarian function [4]. Excessive ROS accumulation disrupts cellular redox homeostasis, induces granulosa cell apoptosis, and compromises mitochondrial membrane potential (MMP), ultimately leading to a deterioration in oocyte quality. Furthermore, redox imbalance may exacerbate ovarian dysfunction by interfering with key signaling pathways.

The Nrf2/HO-1 signaling pathway functions as a fundamental endogenous antioxidant defense system, playing a crucial role in maintaining redox balance and regulating responses to oxidative stress [5]. Dysfunction of this pathway has been associated with accelerated cellular senescence, increased apoptosis, and impaired ovarian function [6]. Pharmacological or genetic activation of Nrf2/HO-1 signaling has been shown to enhance cellular resistance to oxidative damage, thereby mitigating age-related cellular decline and dysfunction.

Kidney-tonifying Chinese herbal medicine has shown significant clinical efficacy in treating infertility among women of advanced maternal age [7]. Erzhi Tiangui Formula (EZTG), a traditional Chinese herbal prescription consisting of ten medicinal herbs (Table 1), is traditionally used to nourish kidney qi and yin according to Traditional Chinese Medicine (TCM) theory [2]. Previous studies have shown that EZTG improves embryo quality in advanced-age patients [8] and increases fertilization rates in patients with diminished ovarian reserve (DOR) and kidney deficiency syndrome undergoing IVF cycles [9]. Additionally, Lian et al. [10] reported that the EZTG formula may downregulate the expression of the pro-apoptotic factor Bad, thereby inhibiting granulosa cell apoptosis and improving IVF outcomes in patients. Nevertheless, the precise mechanisms underlying its reproductive benefits remain to be fully elucidated.

**Table 1:** Herbal components of the EZTG formula

Chinese Name	Botanical Name (Latin)	Medicinal Part Used
Tusizi	*Cuscuta chinensis* Lam.	Seed
Nvzhenzi	*Ligustrum lucidum* Ait.	Fruit
Mohanlian	*Eclipta prostrata* (L.) L.	Whole plant
Gouqizi	*Lycium barbarum* L.	Fruit
Shudihuang	*Rehmannia glutinosa* (Gaertn.) DC.	Prepared root tuber
Danggui	*Angelica sinensis* (Oliv.) Diels	Root
Baishao	*Paeonia lactiflora* Pall.	Root
Chuanxiong	*Ligusticum chuanxiong* Hort.	Rhizome
Xiangfu	*Cyperus rotundus* L.	Rhizome
Zhigancao	*Glycyrrhiza uralensis* Fisch.	Processed root and rhizome

*EZTG,* Erzhi Tiangui

Our previous research demonstrated that EZTG enhances the expression of the antioxidant enzyme SOD in follicular fluid [11], suppresses ROS and Caspase-3 levels in granulosa cells of patients with endometriosis-related infertility [12], and increases Mfn2 expression and mitochondrial membrane potential (MMP) in granulosa cells of advanced-age patients, thereby maintaining ATP stability and improving oocyte quality [13]. However, these studies have primarily focused on its effects on oxidative stress markers within granulosa cells, with limited exploration of its upstream and downstream regulatory targets. Therefore, further investigation into the precise mechanisms by which EZTG enhances oocyte quality is of significant scientific importance.

Building on our previous findings and recognizing the pivotal role of the Nrf2/HO-1 signaling pathway in oxidative stress regulation, this study is the first to propose that EZTG mitigates oxidative stress and protects ovarian function by modulating Nrf2/HO-1 signaling. We hypothesize that EZTG activates this pathway to upregulate antioxidant enzyme expression, thereby reducing oxidative damage in granulosa cells, improving MMP, and inhibiting granulosa cell apoptosis. These effects may ultimately enhance oocyte quality and increase the number of high-quality embryos. This study aims to elucidate the potential mechanisms of a kidney-tonifying herbal medicine like EZTG, providing a scientific basis for further research into infertility in women of advanced maternal age.

## Methods and material

### Revised euthanasia and ethics statement

All animal procedures were conducted in accordance with the National Institutes of Health Guidelines for the Care and Use of Laboratory Animals and were approved by the IACUC of the Reproductive Hospital Affiliated to Shandong University (Approval No. 2022-73).

### Animal study protocol

Six- to eight-week-old (young) and eight-to nine-month-old (aged) female C57BL/6J mice (SPF grade) were obtained from Jinan Pengyue Experimental Animal Co., Ltd. (license No. SCXK(LU)20220006). Following a 7-day acclimation period under controlled conditions (room temperature: 18–22 °C; humidity: 40–60%; 12-h light/dark cycle) with ad libitum access to standard chow and water, estrous cycles were monitored daily for two weeks. A total of 100 mice exhibiting regular cyclicity were enrolled in the study. These animals were divided into five experimental groups: (1) a young control group (YC, *n* = 20) consisting of 6-8-week-old mice, and (2) four aged groups (*n* = 20 each) comprising an aged control (AC) and three treatment groups (low-, medium-, and high-dose EZTG) established by random number table allocation of 8-9-month-old mice.

Dosages were determined through human-to-mouse dose conversion based on body surface area normalization [14]. The standard adult human dose (60 kg) was converted to mouse equivalents (35 g), yielding a daily EZTG dose of 2.86 g/kg (0.1 g/mouse), equivalent to the clinical human dose. Preliminary experiments established the median effective dose (ED50) and maximum tolerated dose (MTD) of EZTG, which informed our final dose selection: low-dose (1.4 g/kg, ½ ED50), medium-dose (5.6 g/kg, 2×ED50), and high-dose (22.4 g/kg, 8×ED50) groups.

**Fig. 1:**
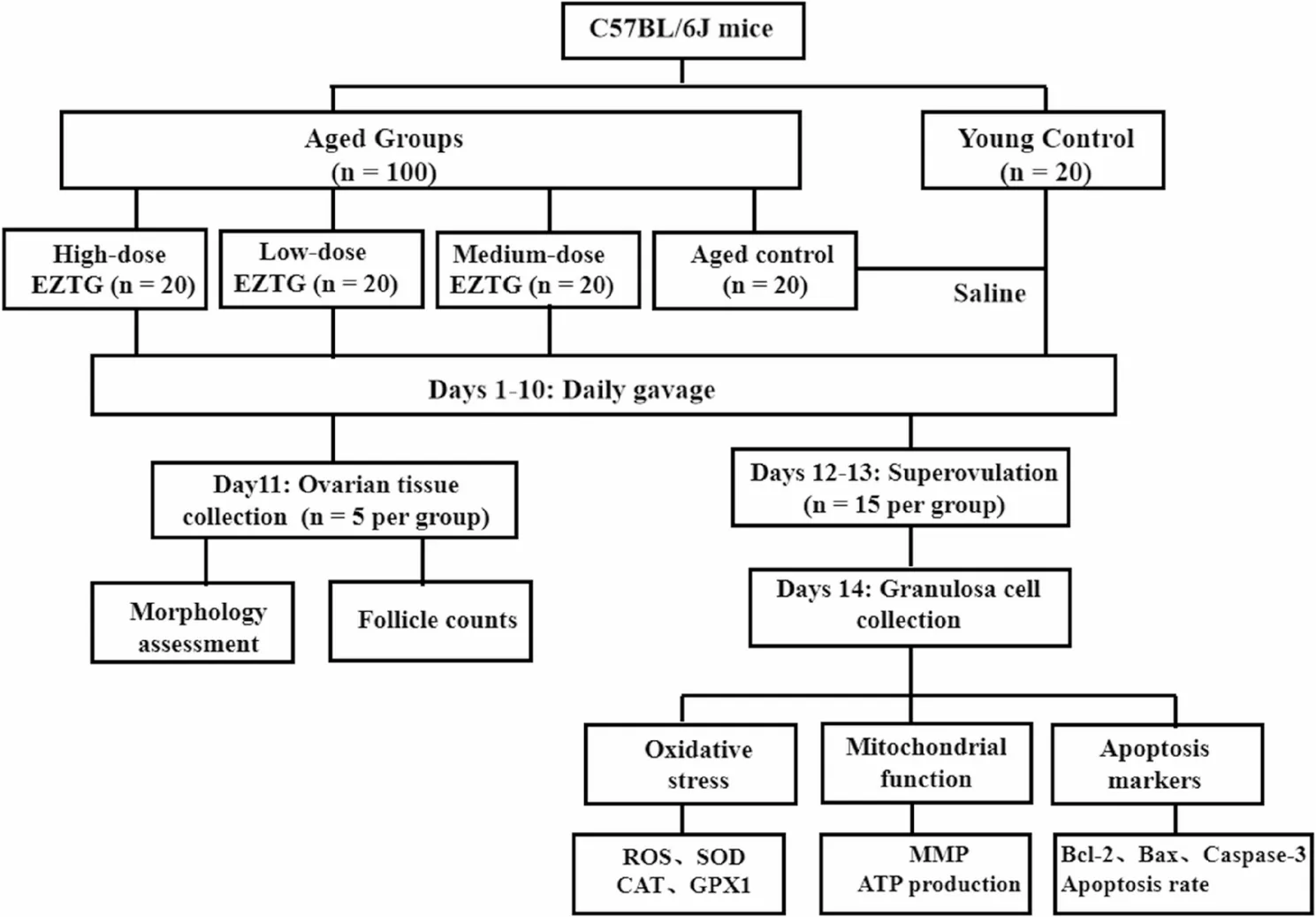
Schematic diagram of the experimental design

The experimental protocol is summarized in Fig. 1. All mice received daily gavage administration at 11:00 AM for 10 consecutive days. Treatment groups were administered varying doses of EZTG, while control groups received equivalent volumes of physiological saline. Estrous cycles were monitored throughout the intervention period. On day 11, five mice per group were euthanized for initial ovarian assessment. Blood samples were collected for serum AMH measurement. Ovarian tissues were then harvested for histological evaluation (HE staining for follicular counts). The remaining animals received ovarian stimulation with human menopausal gonadotropin (hMG 15 IU, Livzon Pharmaceutical, H10940097) followed by human chorionic gonadotropin (hCG 12 IU, Livzon Pharmaceutical, H44020673) 48 h later, and were euthanized 14 h post-hCG administration for subsequent analysis.

### Granulosa cell isolation procedure

Following collection, ovaries were immediately placed in pre-warmed DMEM/F12 medium supplemented with 1% penicillin-streptomycin for granulosa cell isolation. The isolation procedure involved: (1) follicular puncture to release granulosa cells, (2) filtration through a 40 μm cell strainer to remove ovarian debris, (3) centrifugation at 200 × g for 5 min, and (4) two washes with DPBS. The cell pellet was then resuspended in DMEM/F12 containing 5% FBS and 1% antibiotics, and plated at a density of 1 × 10⁵ cells/well in 12-well plates. Cells were maintained at 37 °C in a 5% CO₂ incubator until reaching 90% confluence, at which point the medium was replaced with serum-free DMEM/F12 containing 0.1% BSA for subsequent experimental use.

### Histopathology

Ovarian tissues were fixed in 4% paraformaldehyde for 48 h, followed by dehydration, paraffin embedding, and sectioning at 4 μm thickness. After deparaffinization and rehydration, the sections were stained with hematoxylin for 5–10 min, differentiated in acid alcohol, and counterstained with eosin for 2 min. Subsequently, the slides were dehydrated through a graded ethanol series, cleared in xylene, and mounted for microscopic examination. The morphology of the ovary and the numbers of primordial, primary, secondary, and antral follicles were observed under a microscope.

### Measurement of ROS in granulosa cells

Intracellular ROS levels in granulosa cells labeled with DCFH-DA were measured using a fluorescence microplate reader with excitation at 488 nm and emission at 525 nm, following the manufacturer’s instructions (S0033S, Beyotime Biotechnology, China) .

### GSH measurement in granulosa cells by ELISA

Granulosa cell lysates were prepared using RIPA lysis buffer, and GSH content was measured using an ELISA kit (S0131, Beyotime, China) according to the manufacturer’s instructions.

### Mitochondrial membrane potential (MMP) assay

MMP was assessed using JC-1 staining. Granulosa cells or KGN cells were harvested, incubated with JC-1 working solution (Beyotime Biotechnology, China) at 37 °C for 20 min, and washed twice with JC-1 buffer. Samples were then analyzed by flow cytometry (BD Biosciences, USA). The ratio of red/green fluorescence intensity was calculated to reflect MMP changes. All procedures were performed according to the manufacturer’s instructions.

### ATP measurement in granulosa cells

ATP levels in granulosa cell lysates were measured using a luminometer in accordance with the manufacturer’s instructions (S0026, Beyotime Biotechnology, China).

### Western blot analysis

Proteins were extracted from granulosa cells using RIPA lysis buffer (Beyotime, China, Cat# P0013B) and quantified with a BCA kit (Solarbio, China, Cat# PC0020). The extracted proteins were separated by 10% SDS-PAGE and transferred onto PVDF membranes (Millipore, USA, Cat# IPVH00010). The membranes were blocked with 5% non-fat milk in TBST for 1 h at room temperature, followed by overnight incubation at 4 °C with primary antibodies. The primary antibodies used were as follows: Nrf2 (Proteintech, USA, Cat# 16396-1-AP, dilution 1:1000), HO-1 (Proteintech, USA, Cat# 10701-1-AP, dilution 1:2000), Keap1 (Proteintech, USA, Cat# 10503-2-AP, dilution 1:1000), SOD1 (Proteintech, USA, Cat# 10269-1-AP, dilution 1:1000), CAT (Proteintech, USA, Cat# 21260-1-AP, dilution 1:1000), Bcl-2 (Proteintech, USA, Cat# 26593-1-AP, dilution 1:500), Bax (Proteintech, USA, Cat# 50599-2-Ig, dilution 1:500), and cleaved Caspase-3 (Proteintech, USA, Cat# 25546-1-AP, dilution 1:500). After washing, the membranes were incubated with HRP-conjugated secondary antibody (Beyotime, China, Cat# P0023D, dilution 1:5000) for 1 h at room temperature. Protein bands were visualized using a ChemiDoc Touch Imaging System (Bio-Rad, USA, Cat# 1708370) with an ECL detection kit (Millipore, USA, Cat# WBKLS0500).

### Quantitative real-time PCR (qRT-PCR)

Total RNA was extracted from granulosa cells using TRIzol reagent (1000 µl TRIzol per 10^7 cells). Reverse transcription was performed according to the manufacturer’s protocol. Real-time PCR was conducted using HiScript II Q RT SuperMix for qPCR (Vazyme, United States) and the A100 PCR system. The primer sequences used for real-time PCR (qPCR) are listed in Table 2.The data were expressed as fold changes in mRNA levels relative to the control, calculated using the 2^−∆∆Ct^ method [15].

**Table 2:** Primer sequences for RT-PCR

Gene Name	Primer Sequence (5’→3’)
Mus-Bcl-2-F	GGATTGTGGCCTTCTTTGAGTTC
Mus-Bcl-2-R	CTTCAGAGACAGCCAGGAGAAAT
Mus-BAX-F	GCTACAGGGTTTCATCCAGGATC
Mus-BAX-R	TGCTGTCCAGTTCATCTCCAATT
Mus-Caspase-3-F	CAGCCAACCTCAGAGAGACATT
Mus-Caspase-3-R	TTTCAGTTCAACAGGCCCATTTG
Mus-Nrf2-F	TGGCAGAGACATTCCCATTTGTA
Mus-Nrf2-R	CTTGCTCCATGTCCTGCTCTATG
Mus-HO-1–134 F	GACAGCCCCACCAAGTTCAA
Mus-HO-1-134R	AGCTCCTCAAACAGCTCAATGT
Mus-Keap1-F	ATCCAGAGAGGAATGAGTGGCG
Mus-Keap1-R	TCAACTGGTCCTGCCCATCGTA
Mus-actin-154 F	GGCTGTATTCCCCTCCATCG
Mus-actin-154R	CCAGTTGGTAACAATGCCATGT

### KGN cell culture and treatment

KGN human granulosa cells were cultured in DMEM/F12 medium supplemented with 10% fetal bovine serum (FBS) and 1% penicillin-streptomycin at 37 °C in a 5% CO₂ incubator. For oxidative stress induction, cells were treated with 200 µM H₂O₂ for 24 h. EZTG-containing serum was prepared as described previously and added at indicated concentrations (low, medium, high) 2 h prior to H₂O₂ exposure.

### Nrf2 siRNA knockdown

KGN cells were transfected with Nrf2-specific siRNA (si-Nrf2) or negative control siRNA (si-NC) using Lipofectamine RNAiMAX reagent according to the manufacturer’s instructions. After 48 h of transfection, cells were treated with EZTG-containing serum and/or H₂O₂ as indicated. Knockdown efficiency was confirmed by Western blot analysis.

### Apoptosis assay

Apoptosis rate was determined using an Annexin V-FITC/PI double staining kit. KGN cells were collected, washed with PBS, and resuspended in binding buffer. Cells were incubated with Annexin V-FITC and PI for 15 min at room temperature in the dark, followed by flow cytometry analysis (BD Biosciences, USA). Data were analyzed using FlowJo software.

### Statistical analysis

All data were analyzed using SPSS 25.0 (IBM, USA) and GraphPad Prism 8.0 (GraphPad Software, USA). Sample sizes are specified in figure legends and were consistent across groups. Normality and variance homogeneity were evaluated by Shapiro-Wilk and Levene’s tests, respectively. Normally distributed data with homogeneous variance were presented as mean ± SD/SEM and analyzed by one-way ANOVA followed by Tukey’s HSD post-hoc test; for normally distributed data with heterogeneous variance, Welch’s ANOVA with Games-Howell post-hoc test was used. Non-normally distributed data were assessed via Kruskal-Wallis test with Dunn’s post-hoc test and expressed as median (IQR). A *P* value < 0.05 was considered statistically significant, and all tests were two-tailed.

## Results

### (I)EZTG ameliorates estrous cycle dysfunction in aged mice

**Fig. 2:**
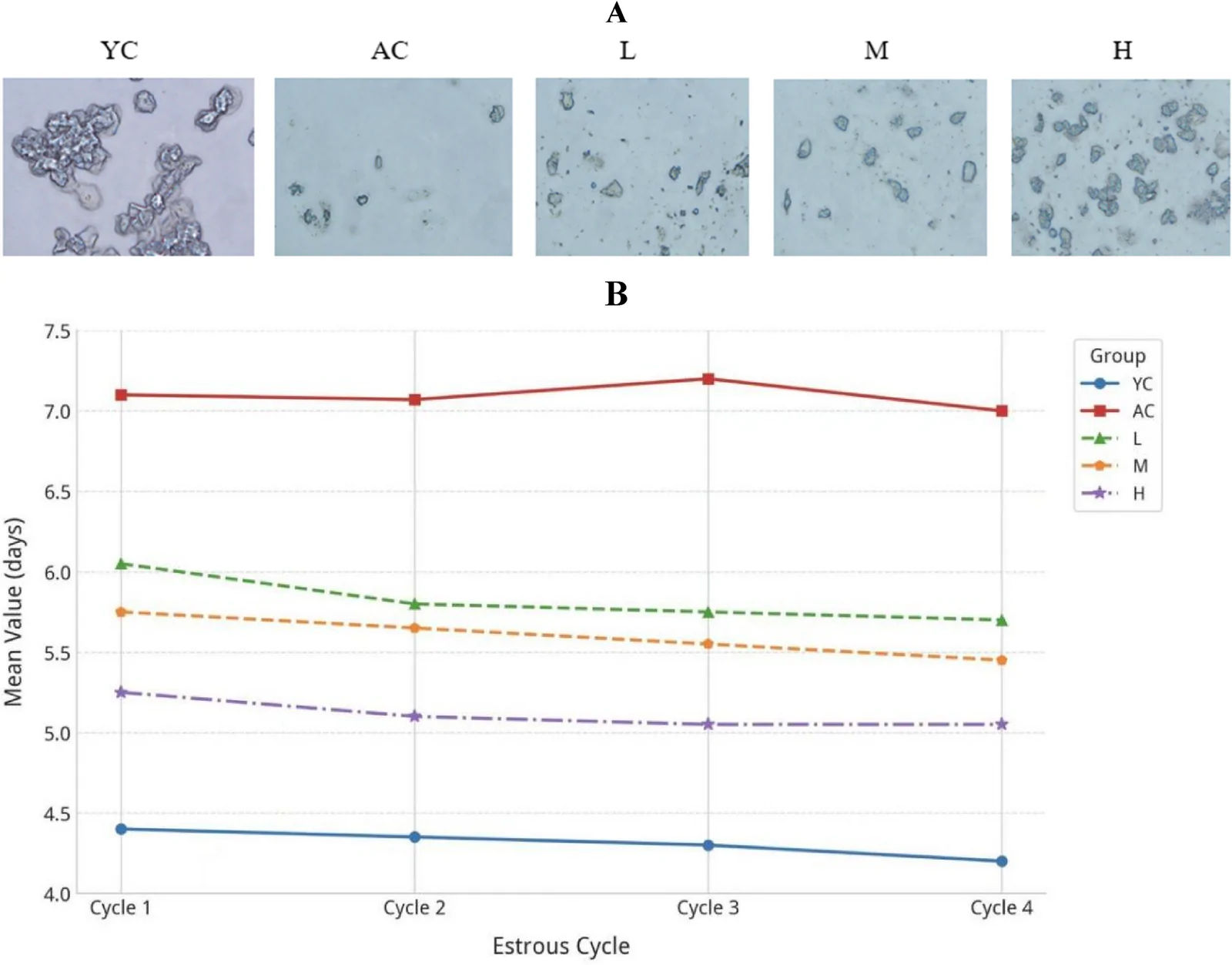
Vaginal cytology changes during the estrous cycle in mice. **A** Representative images of vaginal smears from different groups. **B** Quantitative analysis of cycle length (days) over four consecutive cycles. Data are presented as mean ± SEM (*n* = 20 per group). **P < 0.01, ***P < 0.001 vs. AC group

The YC group exhibited regular estrous cycles (4–5 days), while AC mice showed marked cycle disruption, with prolonged (6–9 days) and irregular phases; vaginal cytology also revealed fewer keratinized epithelial cells and increased leukocytes and mucus in the AC group (Fig. 2A). EZTG intervention dose-dependently reversed these age-related abnormalities, with the high-dose group showing the strongest improvement in cycle regularity and phase synchrony. Quantitative analysis of cycle length over four consecutive cycles confirmed that AC mice had significantly longer cycles than YC mice, and EZTG treatment shortened cycle length toward normal levels (Fig. 2B). Additionally, the cycle irregularity rate (defined as the percentage of cycles lasting > 6 days) was 0% in YC mice but 90% in AC mice (***P < 0.001 vs. YC), and medium- and high-dose EZTG significantly lowered this irregularity rate compared with the AC group (#*P* < 0.05, ##*P* < 0.01; Table 3).

**Table 3:** Effect of EZTG on estrous cycle regularity in aged mice

Group	Number (*n*)	Normal Cycles (*n*)	Prolonged Cycles(*n*)	Cycle Irregularity Rate (%)
YC	20	20	0	0
AC	20	2	18	90***
L	20	7	13	65
M	20	10	10	50^#^
H	20	12	8	40^##^

Group abbreviations: YC, young control; AC, aged control; L, low-dose EZTG; M, medium-dose EZTG; H, high-dose EZTG. These abbreviations apply to all tables and figures in this study. Prolonged estrous cycle is defined as a cycle lasting more than 6 days. Cycle disorder rate = (number of prolonged cycles / total number of cycles) × 100%. ***P < 0.001 vs. YC group; #*P*<0.05, ##*P*<0.01 vs. AC group

### (II) EZTG improves ovarian histomorphology, follicle development and ovarian reserve in aged mice

**Fig. 3:**
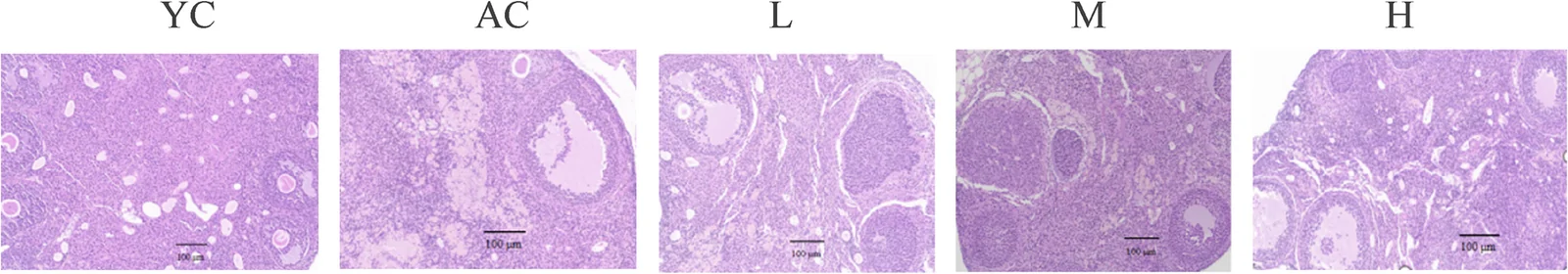
Representative histological images of ovarian sections from different groups of mice (H&E staining; scale bar = 100 μm). The AC group shows disorganized granulosa cell layers, expanded intercellular spaces, and increased atretic follicles (black arrows). EZTG treatment dose-dependently restored follicular structure and reduced atresia. Quantitative analysis of follicle counts at different stages is presented in Table 4

Histological examination of ovarian sections(H&E staining)showed intact ovarian architecture and well-organized follicles in YC mice, while AC mice exhibited severe ovarian damage, including disorganized granulosa cell layers, widened interstitial spaces, and increased atretic follicles (black arrows, Fig. 3). EZTG treatment dose-dependently reversed these pathological changes and reduced follicular atresia.

**Table 4:** Effects of EZTG on ovarian follicle counts at different developmental stages

Group	Primordial Follicles	Primary Follicles	Secondary Follicles	Antral Follicles	Atretic Follicles
YC	9.15 ± 1.94	6.65 ± 0.95	7.58 ± 1.01	8.7 ± 1.29	1.88 ± 0.79
AC	2.7 ± 1.09^***^	2.0 ± 0.88^***^	2.48 ± 1.06^***^	2.73 ± 1.13^***^	6.05 ± 1.22^***^
L	3.23 ± 0.66	2.28 ± 0.68	3.53 ± 0.85^###^	2.98 ± 0.66	5.5 ± 0.68^#^
M	5.23 ± 1.12^###^	3.98 ± 1.03^###^	5.83 ± 1.50^###^	5.53 ± 0.64^###^	4.8 ± 0.91^###^
H	7.7 ± 1.04^###^	5.93 ± 1.19^###^	7.0 ± 0.85^###^	6.73 ± 0.82^###^	3.75 ± 1.10^###^

Data are presented as mean ± SD (*n* = 5 per group). Follicle classification was based on standard mouse ovarian histology. ***P < 0.001 vs. YC group; #*P* < 0.05, ###*P* < 0.001 vs. AC group

Follicle quantification (Table 4) further confirmed that AC mice had significantly fewer viable follicles at all stages and more atretic follicles than YC mice (all ***P < 0.001). EZTG treatment dose-dependently increased viable follicle numbers and decreased atretic follicles, with marked effects detected in the medium- and high-dose groups (#*P* < 0.05, ###*P* < 0.001 vs. AC).

**Fig. 4:**
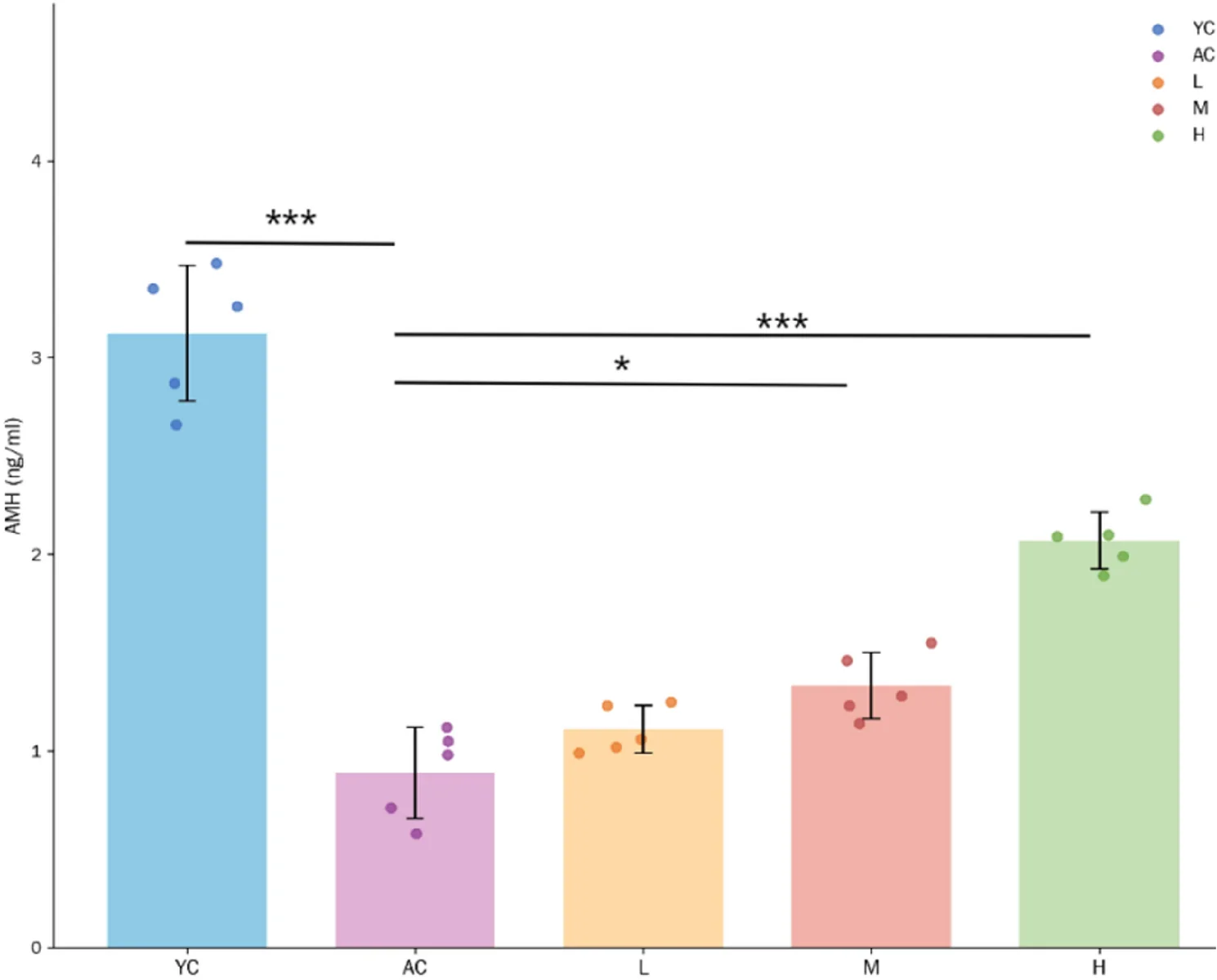
Serum AMH levels in different experimental groups. Data are presented as mean ± SD (*n* = 5 per group). *P < 0.05, ***P < 0.001 vs. AC group

Serum AMH, a core biomarker of ovarian reserve, was significantly lower in AC mice than in YC mice (Fig. 4). EZTG treatment elevated AMH levels in a clear dose-dependent manner, with significant improvements observed in the medium- and high-dose groups (*P < 0.05, ***P < 0.001 vs. AC). These findings are consistent with the follicle count data, confirming that EZTG effectively preserves ovarian reserve in aged mice.

### (III) EZTG modulates oxidative stress markers and Nrf2/HO-1 pathway in granulosa cells

**Fig. 5:**
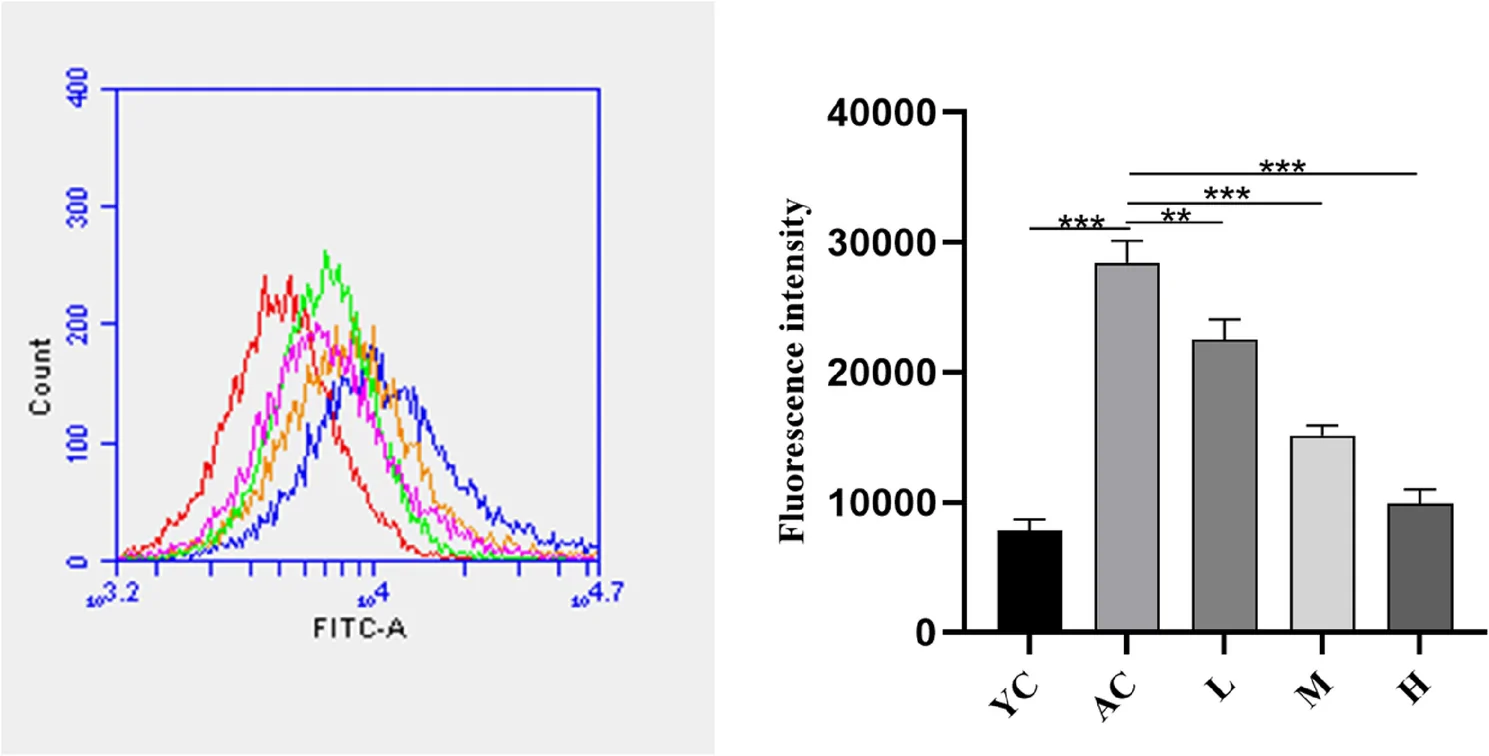
Effects of EZTG on ROS levels in granulosa cells of mice. **A** Representative flow cytometry histograms showing ROS fluorescence intensity (FITC-A channel) in granulosa cells from each group. **B** Quantitative analysis of ROS fluorescence intensity. Data are presented as mean ± SD (*n* = 5 per group). ***P < 0.001, **P < 0.01 vs. AC group

As shown in Fig. 5, the AC group exhibited significantly higher ROS levels than the YC group (*P* < 0.001), indicating age-related oxidative stress. Treatment with low-, medium-, and high-dose EZTG reduced ROS levels in a dose-dependent manner, with the most pronounced effect observed in the high-dose group.

**Fig. 6:**
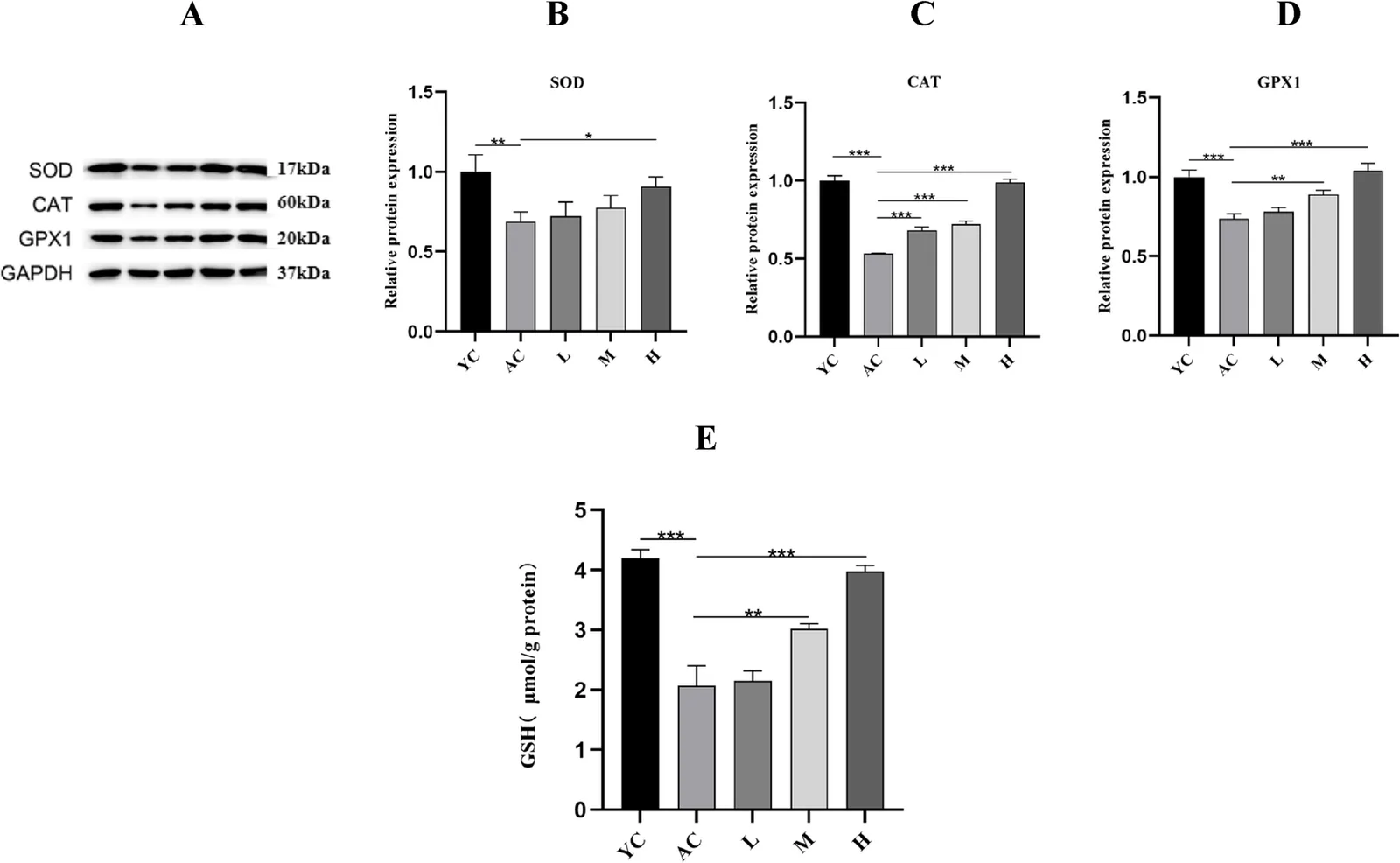
Effects of EZTG on oxidative stress markers in granulosa cells of mice. **A** Representative Western blot bands of SOD, CAT, and GPX1. **B**-**D** Densitometric quantification of SOD (**B**), CAT (**C**), and GPX1 (**D**) protein levels normalized to GAPDH. **E** GSH content measured by ELISA. Data are presented as mean ± SD (*n* = 5 per group). *P < 0.05, **P < 0.01, ***P < 0.001 vs. AC group

As shown in Fig. 6, comparative analysis revealed significantly lower protein expression of antioxidant enzymes (SOD, CAT, GPX1) and reduced glutathione (GSH) levels in granulosa cells of AC mice compared with YC mice, indicating impaired antioxidant capacity that likely contributes to ROS accumulation and oxidative damage. EZTG treatment significantly restored SOD levels, with the medium- and high-dose groups showing the greatest efficacy (*P* < 0.05 and *P* < 0.01 vs. AC, respectively). In addition, EZTG dose-dependently increased CAT and GSH levels (all *P* < 0.01 vs. AC) and significantly upregulated GPX1 expression, particularly in the medium- and high-dose groups (*P* < 0.01 and *P* < 0.001 vs. AC, respectively).

**Fig. 7:**
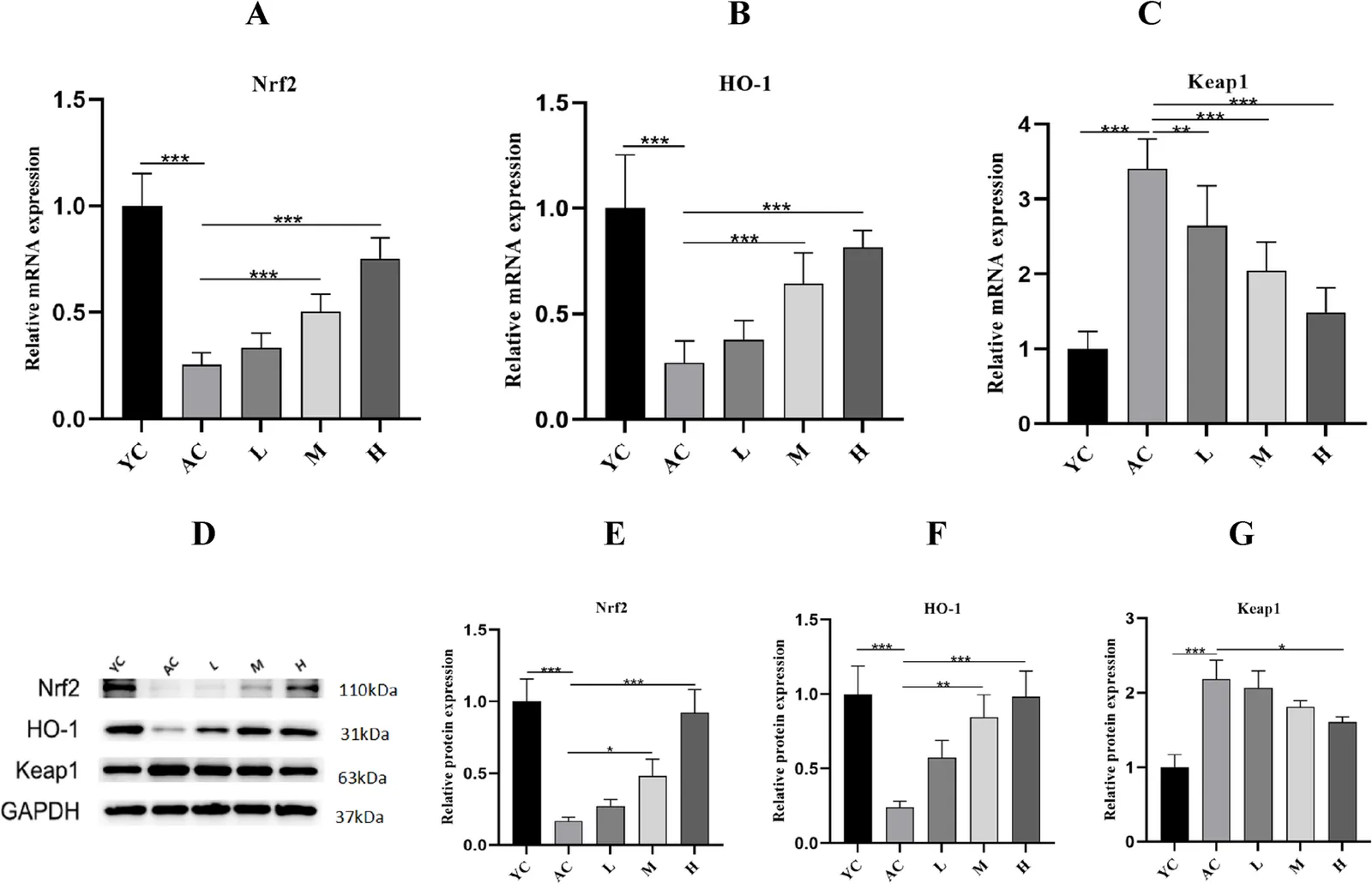
Expression of Nrf2/HO-1 pathway components in granulosa cells from different groups of mice. **A**-**C** Relative mRNA levels of Nrf2 (**A**), HO-1 (**B**), and Keap1 (**C**) measured by RT-qPCR. **D** Representative Western blot bands of Nrf2, HO-1, and Keap1. **E**-**G** Densitometric quantification of Nrf2 (**E**), HO-1 (**F**), and Keap1 (**G**) protein levels, normalized to GAPDH. Data are presented as mean ± SD (*n* = 5 per group). *P < 0.05, **P < 0.01, ***P < 0.001 vs. AC group

As shown in Fig. 7, the mRNA and protein levels of Nrf2, HO-1, and Keap1 in granulosa cells were assessed by RT-qPCR and Western blot, respectively. Compared with the YC group, the AC group exhibited significantly decreased mRNA expression of Nrf2 and HO-1 (both *P* < 0.001), alongside a marked increase in Keap1 mRNA expression (*P* < 0.001). EZTG treatment dose-dependently reversed these changes: Nrf2 and HO-1 mRNA levels were significantly upregulated in all EZTG-treated groups (*P* < 0.001 vs. AC), while Keap1 mRNA expression was downregulated in the medium- (*P* < 0.01) and high-dose groups (*P* < 0.001 vs. AC; Fig. 7A-C). At the protein level, Nrf2 expression was significantly increased in the medium- (*P* < 0.05) and high-dose groups (*P* < 0.001 vs. AC), and HO-1 expression was also elevated in the medium- (*P* < 0.01) and high-dose groups (*P* < 0.001 vs. AC). Conversely, Keap1 protein levels were significantly reduced in the medium- (*P* < 0.05) and high-dose groups (*P* < 0.001 vs. AC; Fig. 7D-G).

### (IV) EZTG inhibits apoptosis-related factors in granulosa cells of aged mice

**Fig. 8:**
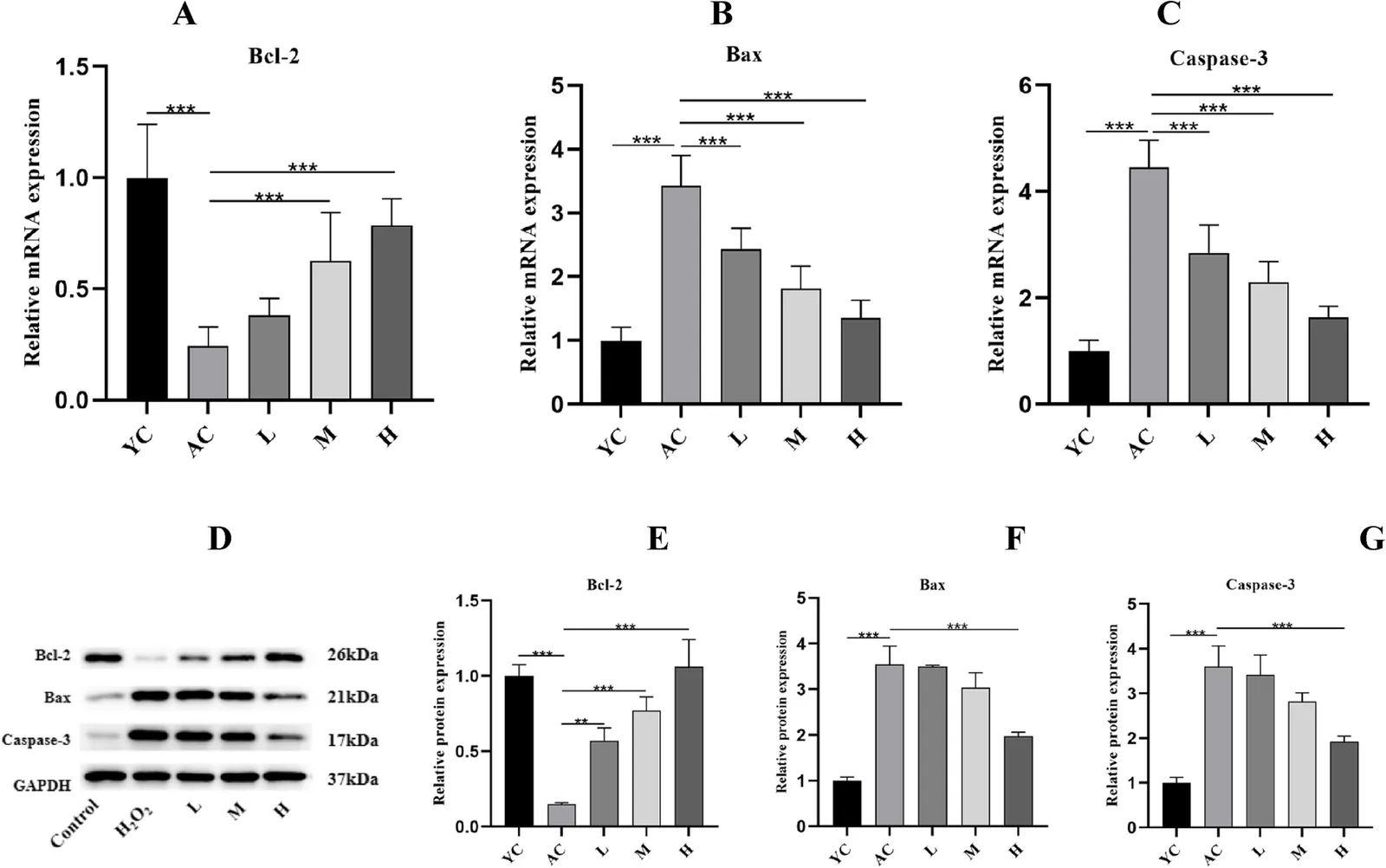
Expression of apoptosis-related factors in granulosa cells from different groups of mice. **A**-**C** Relative mRNA levels of Bcl-2 (**A**), Bax (**B**), and cleaved Caspase-3 (**C**) measured by RT-qPCR. **D** Representative Western blot bands of Bcl-2, Bax, and cleaved Caspase-3. **E**-**G** Quantitative analysis of Bcl-2 (**E**), Bax (**F**), and cleaved Caspase-3 (**G**) protein levels, normalized to GAPDH. Data are presented as mean ± SD (n = 5 per group). *P < 0.05, **P < 0.01, ***P < 0.001 vs. AC group

As shown in Fig. 8, the mRNA expression levels of apoptosis-related factors in granulosa cells were significantly altered among the groups. Specifically, Bcl-2 mRNA expression was markedly decreased in the AC group compared with the YC group, while Bax and cleaved Caspase-3 mRNA levels were significantly increased (all *P* < 0.001; Fig. 8A-C).

EZTG treatment reversed these changes in a dose-dependent manner. Compared with the AC group, the low-, medium-, and high-dose EZTG groups exhibited significant upregulation of Bcl-2 mRNA (*P* < 0.001) and downregulation of Bax and cleaved Caspase-3 mRNA (all *P* < 0.001).

Consistent with the mRNA data, Western blot analysis of protein expression (Fig. 8D-G) demonstrated that Bcl-2 protein levels were significantly increased in EZTG-treated groups (medium and high doses, *P* < 0.01), whereas Bax and cleaved Caspase-3 protein levels were significantly decreased following EZTG treatment (*P* < 0.001).

These findings suggest that EZTG effectively modulates the expression of apoptosis-related genes and proteins in granulosa cells, promoting cell survival by upregulating anti-apoptotic Bcl-2 and downregulating pro-apoptotic Bax and cleaved Caspase-3.

### (V)EZTG ameliorates MMPloss in granulosa cells of aged mice

**Fig. 9:**
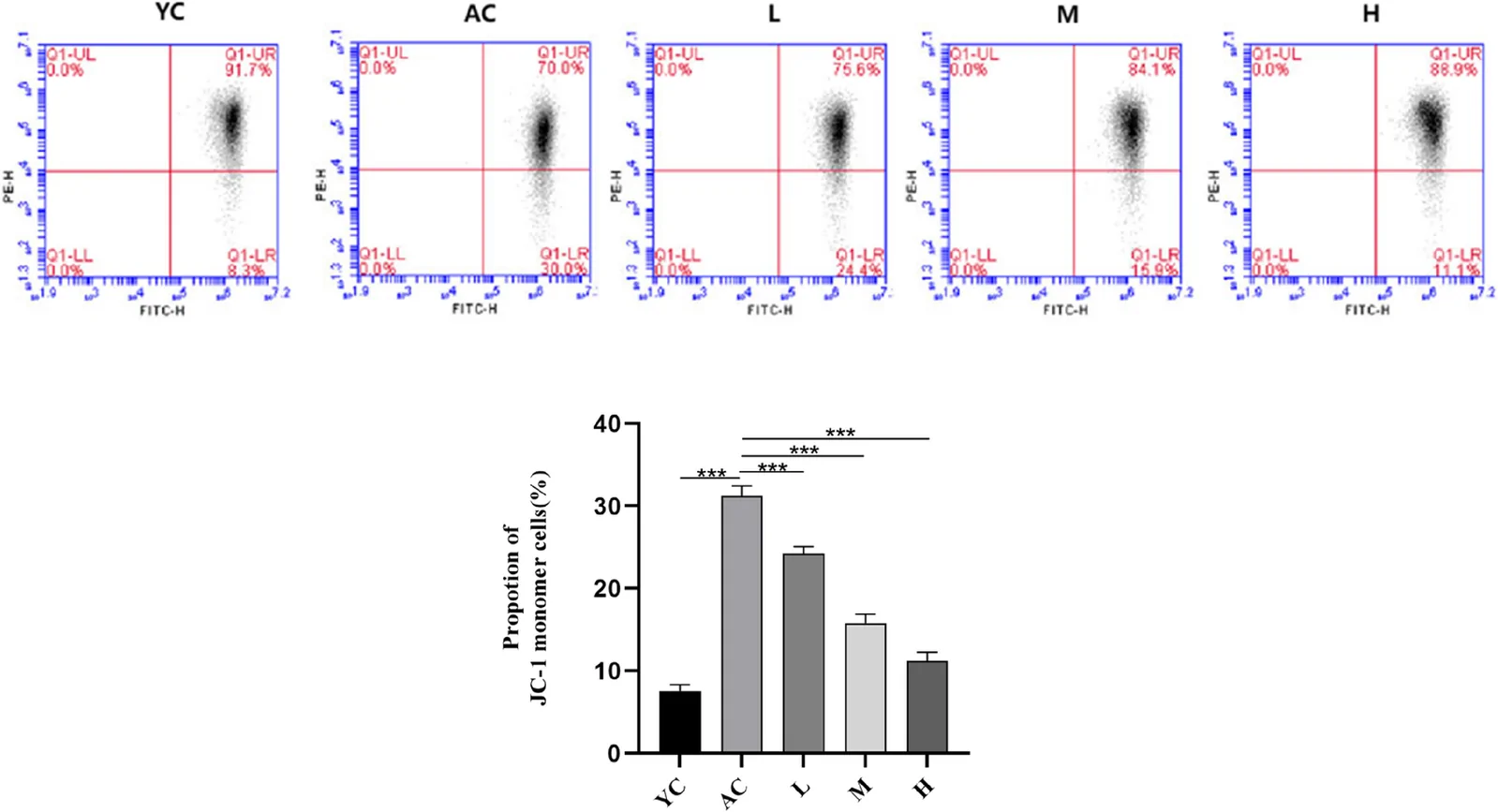
Effects of EZTG on MMP in granulosa cells from different groups of mice. **A** Representative flow cytometry plots of JC-1 staining. The percentage of cells in the Q1-UR quadrant (PE-H⁺FITC-H⁺, JC-1 aggregates) reflects the proportion of cells with high MMP. **B** Quantitative analysis of the proportion of JC-1 monomer cells (green fluorescence). Data are presented as mean ± SD (*n* = 5 per group). ***P < 0.001 vs. AC group

Comparative analysis revealed a significantly higher percentage of JC-1 monomer-positive granulosa cells in AC mice compared to YC mice (*P* < 0.001), indicating age-related mitochondrial depolarization. EZTG treatment dose-dependently reduced the proportion of JC-1 monomers (all *P* < 0.001 vs. AC group), with the high-dose group showing the greatest restoration of MMP (Fig. 9).

### (VI) EZTG improves ATP levels in granulosa cells of aged mice

**Fig. 10:**
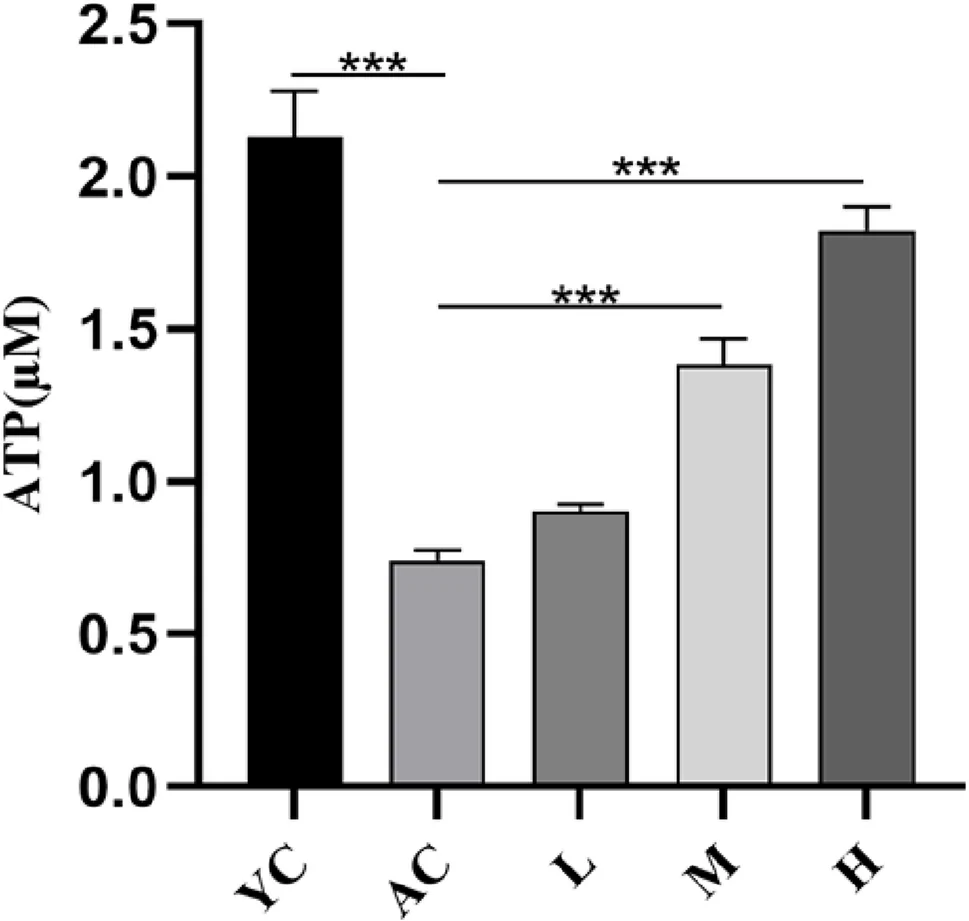
Effects of EZTG on ATP levels in granulosa cells. ATP content was measured by luminescent assay.Data are mean ± SD (*n* = 5 per group). ***P < 0.001 vs. AC group

Compared to the YC group, ATP levels in granulosa cells of the AC group were significantly reduced (*P* < 0.001), suggesting that aging may impair cellular energy metabolism, leading to mitochondrial dysfunction and cellular senescence. Following EZTG intervention, ATP levels in granulosa cells of aged mice were significantly increased in a dose-dependent manner (all *P* < 0.001 vs. AC group). This indicates that EZTG effectively improves energy metabolism in granulosa cells by enhancing ATP levels, thereby alleviating aging-induced functional impairment (Fig. 10).

### (VII)Validation of Nrf2 pathway activation in KGN cells

To further elucidate whether EZTG directly activates the Nrf2 pathway rather than inducing a compensatory stress response, we performed additional mechanistic studies using KGN human granulosa cells under H₂O₂-induced oxidative stress.

### Effects of EZTG on oxidative stress factors in H₂O₂-treated KGN cells

**Fig. 11:**
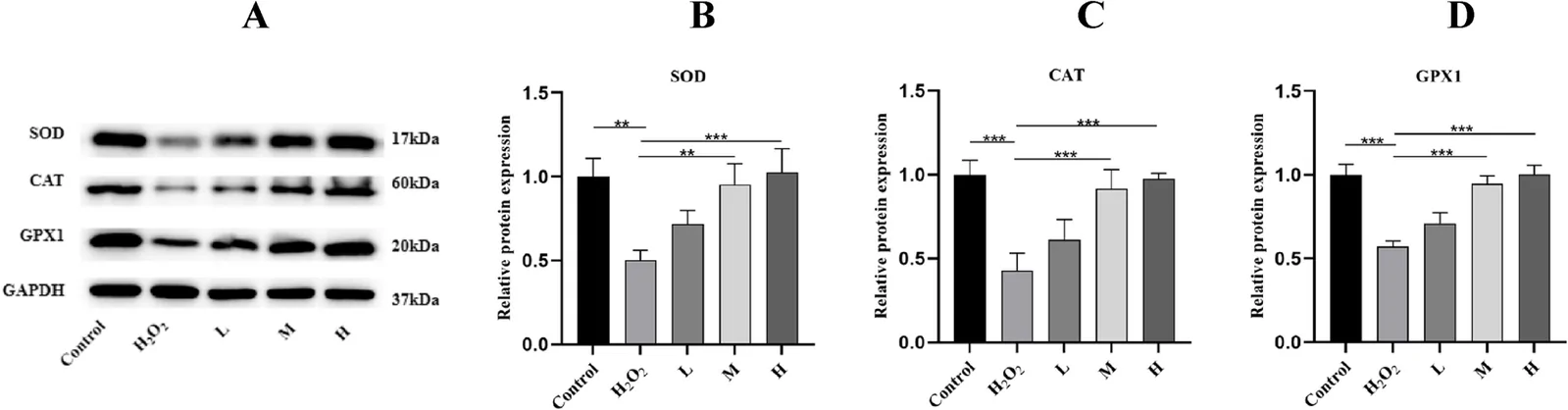
Effects of EZTG on oxidative stress factors in H₂O₂-treated KGN cells. **A** Representative Western blot bands showing protein expression of antioxidant enzymes SOD, CAT, and GPX1 in KGN cells under different treatment conditions. GAPDH was used as a loading control. **B**-**D** Quantitative analysis of SOD (**B**), CAT (**C**), and GPX1 (**D**) protein levels, normalized to GAPDH. Data are presented as mean ± SD from three independent experiments. **P < 0.01, ***P < 0.001 vs. H₂O₂ group. L: H₂O₂+EZTG-L; M:H₂O₂+EZTG-M; H:H₂O₂+EZTG-H. These group definitions apply to Figs. 11, 12 and 13

As shown in Fig. 11, H₂O₂ treatment significantly decreased the protein expression of antioxidant enzymes SOD, CAT, and GPX1 in KGN cells (all *P* < 0.01 vs. control group). EZTG treatment dose-dependently restored the expression of these enzymes (all *P* < 0.01 vs. H₂O₂ group), with higher concentrations exhibiting greater efficacy.

### Effects of EZTG on Nrf2/HO-1 pathway in H₂O₂-treated KGN cells

**Fig. 12:**
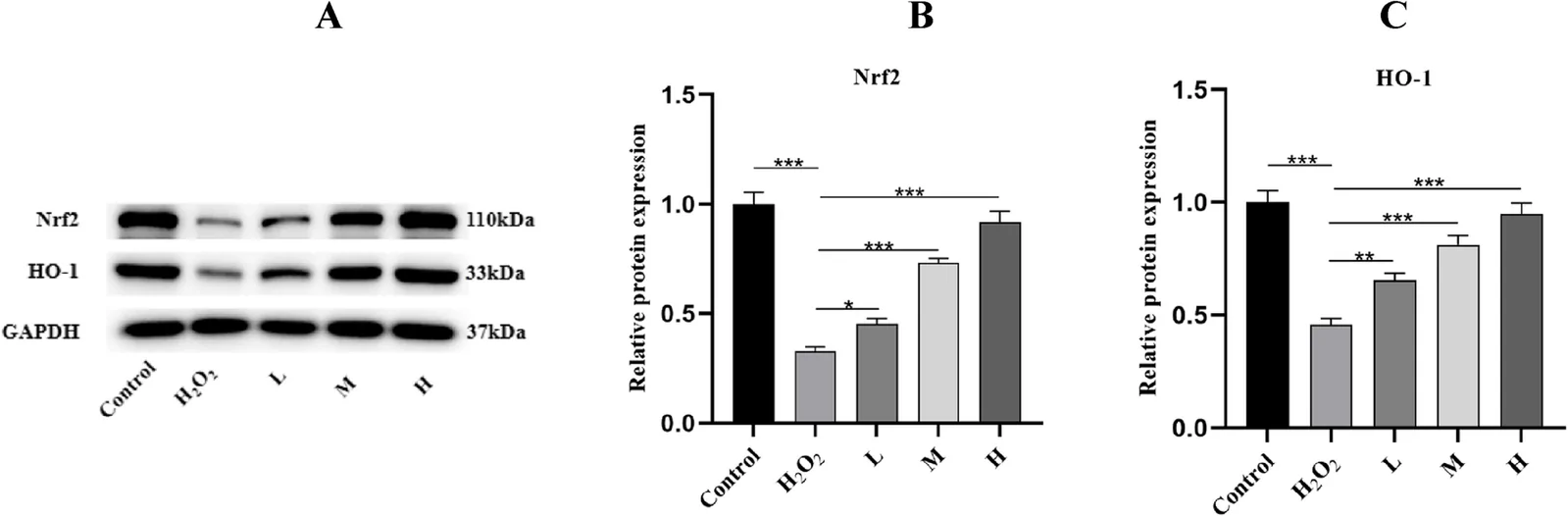
Effects of EZTG on Nrf2 and HO-1 expression in H₂O₂-treated KGN cells. **A** Representative Western blot bands showing protein expression of Nrf2 and HO-1 in KGN cells under different treatment conditions. GAPDH was used as a loading control, with molecular weights indicated on the right. **B**-**C** Quantitative analysis of Nrf2 (**B**) and HO-1 (**C**) protein levels, normalized to GAPDH. Data are presented as mean ± SD from three independent experiments. *P < 0.05, **P < 0.01, ***P < 0.001 vs. H₂O₂ group

Compared with the control group, H₂O₂ treatment markedly reduced the protein expression of Nrf2 and HO-1 (both *P* < 0.001). EZTG treatment significantly upregulated Nrf2 and HO-1 levels in a dose-dependent manner (all *P* < 0.05 vs. H₂O₂ group), with the high-dose group showing the most pronounced effect (Fig. 12).

### Effects of EZTG on apoptosis in H₂O₂-treated KGN cells

**Fig. 13:**
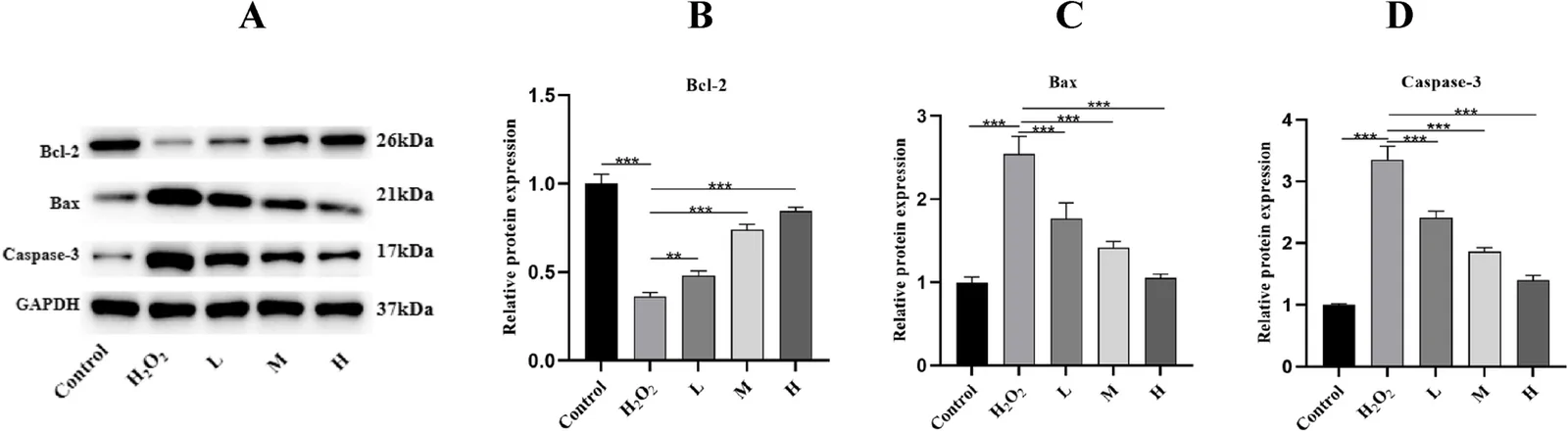
Effects of EZTG on apoptotic factor expression in H₂O₂-treated KGN cells. **A** Representative Western blot bands showing protein expression of Bcl-2, Bax, and cleaved Caspase-3 in KGN cells under different treatment conditions. GAPDH was used as a loading control, with molecular weights indicated on the right. **B**-**D** Quantitative analysis of Bcl-2 (**B**), Bax (**C**), and cleaved Caspase-3 (**D**) protein levels, normalized to GAPDH. Data are presented as mean ± SD from three independent experiments. **P < 0.01, ***P < 0.001 vs. H₂O₂ group

Flow cytometry analysis revealed that H₂O₂ treatment significantly increased the apoptosis rate of KGN cells (*P* < 0.001 vs. control group), which was dose-dependently reversed by EZTG treatment (all *P* < 0.001 vs. H₂O₂ group; Fig. 13A). Consistently, H₂O₂ upregulated pro-apoptotic proteins Bax and cleaved Caspase-3 and downregulated anti-apoptotic Bcl-2 (all *P* < 0.001 vs. control group), while EZTG treatment normalized these changes in a dose-dependent manner (all *P* < 0.001 vs. H₂O₂ group; Fig. 13B).

### Nrf2 knockdown abolishes EZTG-mediated protective effects

**Fig. 14:**
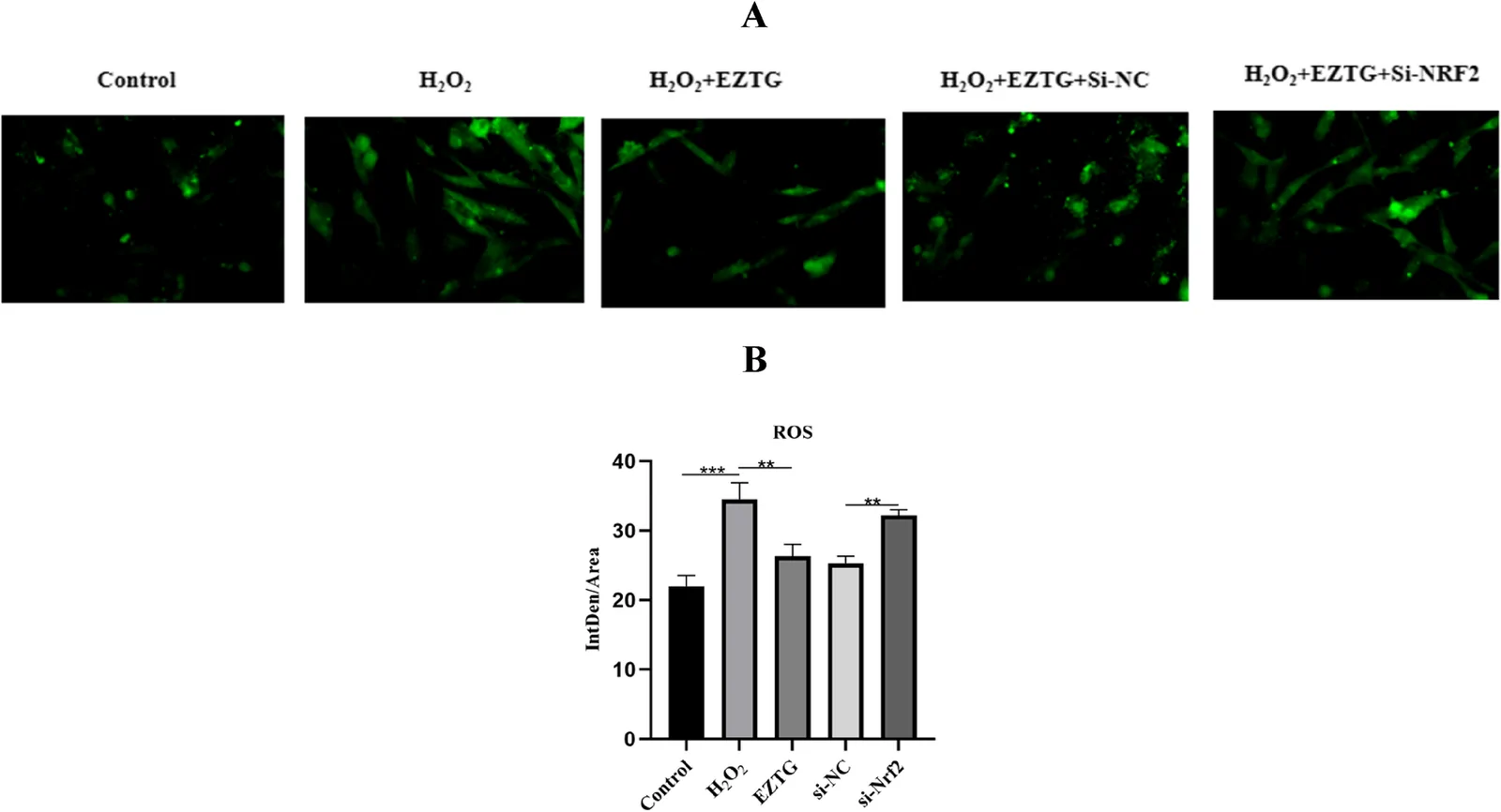
Effects of Nrf2 silencing on EZTG-mediated ROS scavenging in H₂O₂-induced KGN cells. **A** Representative fluorescence images of intracellular ROS levels (green fluorescence) in KGN cells under different treatments. **B** Quantitative analysis of ROS intensity (IntDen/Area).Data are presented as mean ± SD from three independent experiments. **P < 0.01, ***P < 0.001 vs. indicated groups. EZTG: H₂O₂+EZTG; si-NC: H₂O₂+EZTG + si-NC; si-Nrf2: H₂O₂+EZTG + si-Nrf2. These group definitions apply to Figs. 14, 15, 16, 17 and 18

**Fig. 15:**
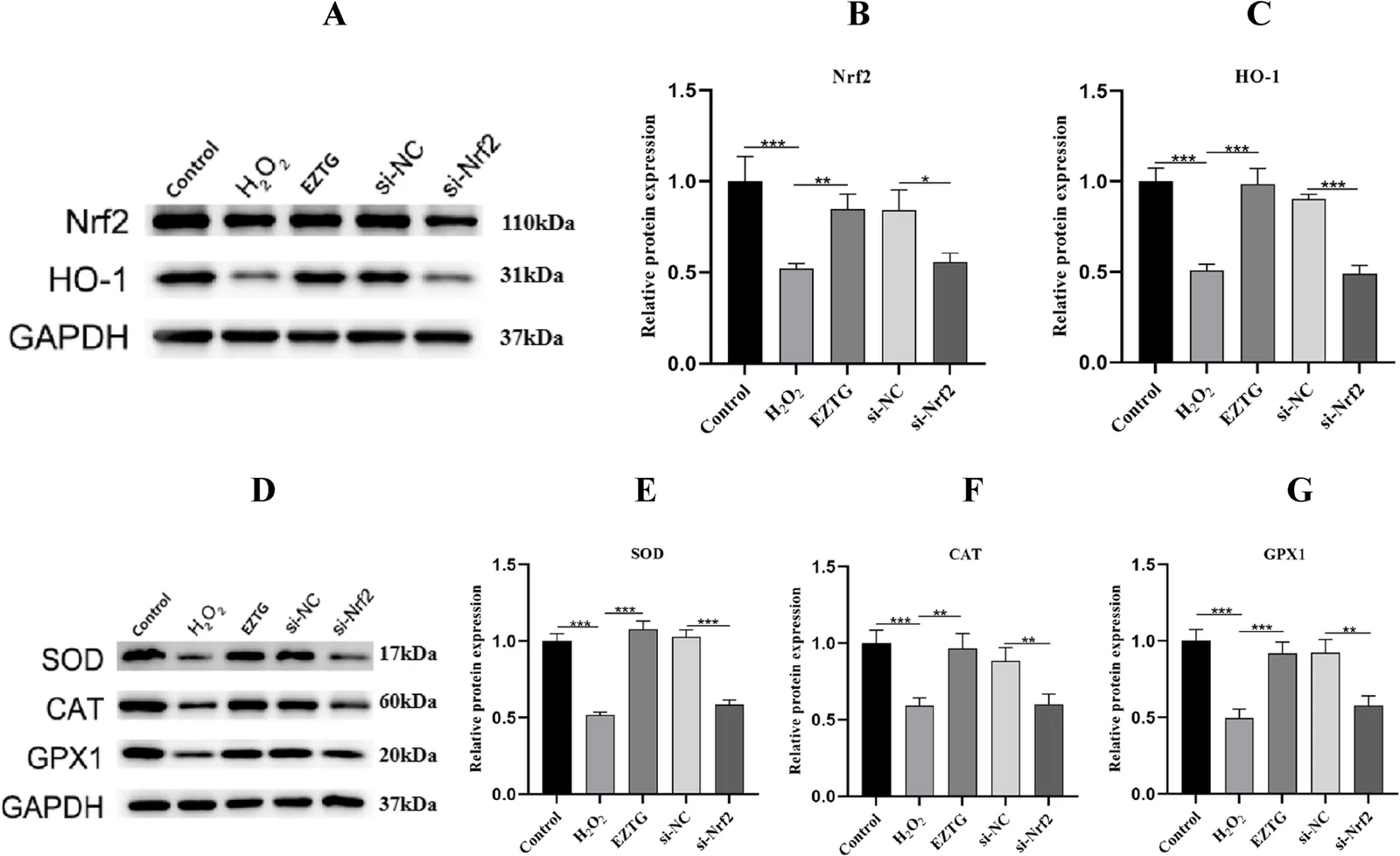
Effects of Nrf2 silencing on EZTG-mediated regulation of the Nrf2/HO-1 pathway and antioxidant enzymes in H₂O₂-induced KGN cells. **A** Representative Western blot bands showing protein expression of Nrf2 and HO-1 in KGN cells under different treatments. **B**, **C** Quantitative analysis of Nrf2 (**B**) and HO-1 (**C**) protein levels, normalized to GAPDH. **D** Representative Western blot bands showing protein expression of SOD, CAT, and GPX1. (E-G) Quantitative analysis of SOD, CAT, and GPX1 protein levels, normalized to GAPDH. Data are presented as mean ± SD from three independent experiments. *P < 0.05, **P < 0.01, ***P < 0.001 vs. indicated groups

**Fig. 16:**
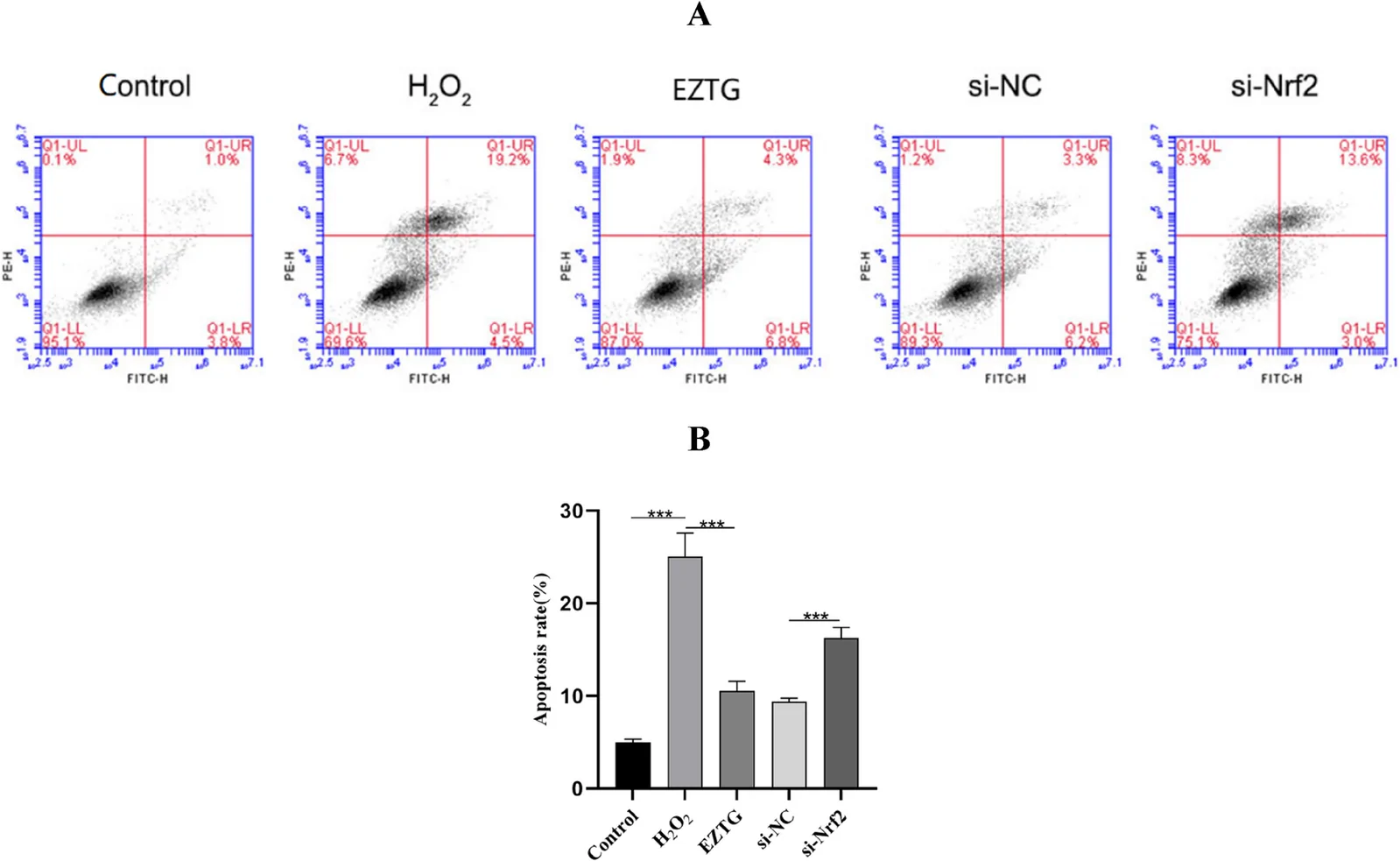
Effects of Nrf2 silencing on EZTG-mediated inhibition of apoptosis in H₂O₂-induced KGN cells. **A** Representative flow cytometry plots of KGN cell apoptosis. **B** Quantification of apoptosis rate. Data are mean ± SD. ***P < 0.001 vs. indicated groups

**Fig. 17:**
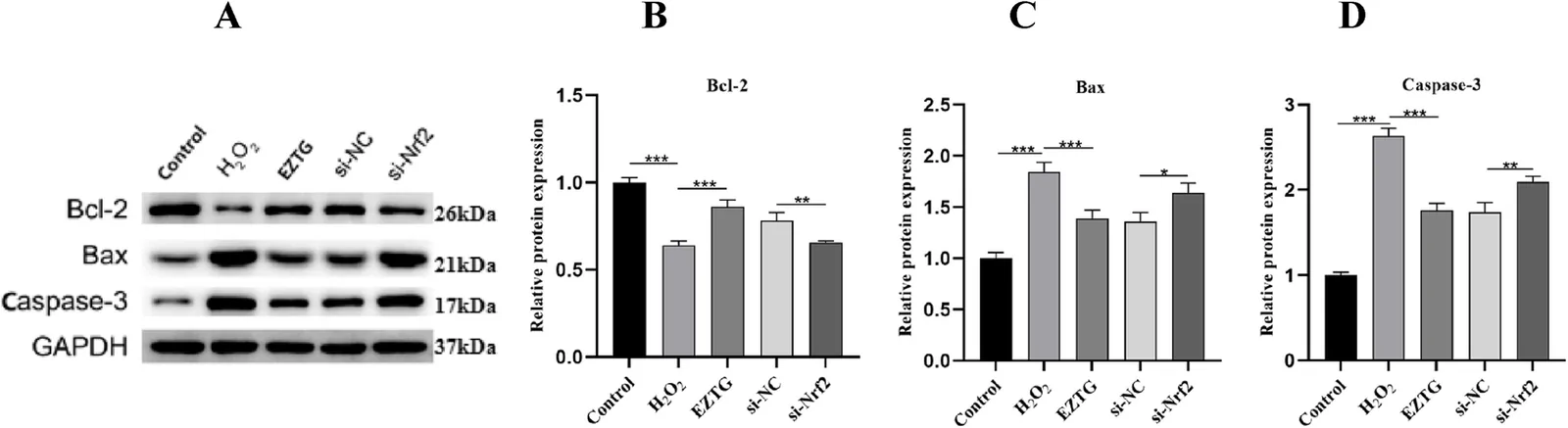
Effects of Nrf2 silencing on EZTG-mediated regulation of apoptosis-related proteins in H₂O₂-induced KGN cells. **A** Representative Western blot bands showing protein expression of Bcl-2, Bax, and cleaved Caspase-3 under different treatments. **B**-**D** Quantitative analysis of Bcl-2 (**B**), Bax (**C**), and cleaved Caspase-3 (**D**) protein levels, normalized to GAPDH. Data are presented as mean ± SD from three independent experiments. *P < 0.05, **P < 0.01, ***P < 0.001 vs. indicated groups

**Fig. 18:**
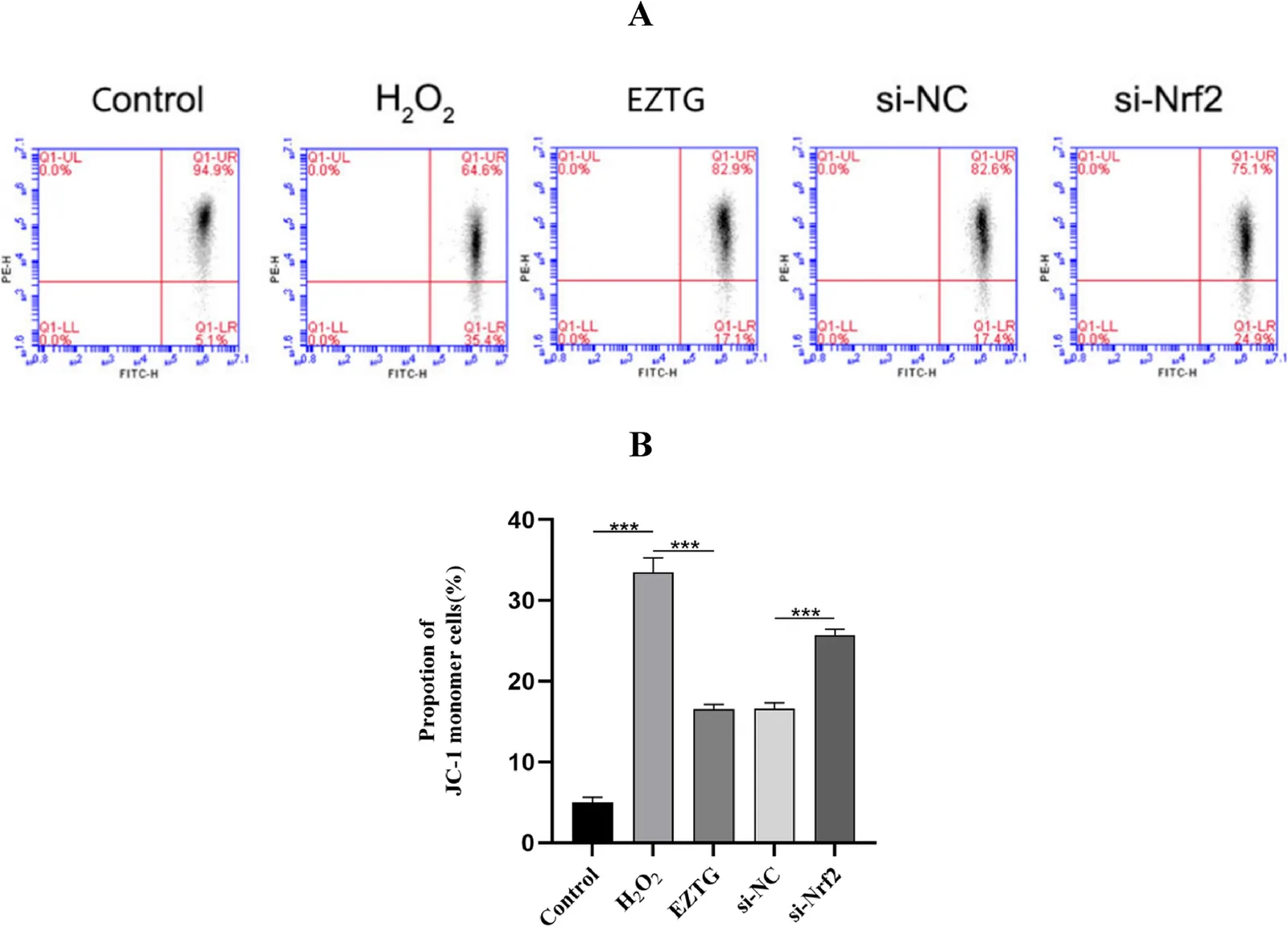
Effects of Nrf2 silencing on EZTG-mediated protection of MMP in H₂O₂-induced KGN cells. **A** Representative flow cytometry plots of JC-1 staining, showing the proportion of cells with high (JC-1 aggregates) and low (JC-1 monomers) MMP. **B** Quantitative analysis of the proportion of JC-1 monomer cells (indicating low MMP). Data are presented as mean ± SD from three independent experiments. ***P < 0.001 vs. indicated groups

To determine whether Nrf2 is functionally required for EZTG’s protective effects, we performed siRNA-mediated Nrf2 knockdown experiments. As shown in Fig. 14, Nrf2 silencing significantly attenuated EZTG-induced ROS scavenging (*P* < 0.01 vs. EZTG group). Furthermore, Nrf2 knockdown abolished the EZTG-mediated upregulation of HO-1, SOD, CAT, and GPX1 (all *P* < 0.05 vs. EZTG group; Fig. 15). The anti-apoptotic effect of EZTG was also reversed by Nrf2 silencing, as evidenced by an increased apoptosis rate (Fig. 16), downregulated Bcl-2, and upregulated Bax and cleaved Caspase-3 (Fig. 17). Additionally, EZTG-mediated restoration of MMP was lost upon Nrf2 knockdown (*P* < 0.001 vs. EZTG group; Fig. 18).

These loss-of-function experiments establish Nrf2 as an essential mediator of EZTG’s antioxidant and cytoprotective effects, providing functional evidence that Nrf2 pathway activation underlies the therapeutic actions of EZTG against oxidative stress-induced granulosa cells damage.

## Discussion

Ovarian aging is a leading cause of infertility in advanced-age women and presents a significant clinical challenge [16]. Current interventions, such as coenzyme Q10 supplementation and growth hormone injections, have yielded limited success, as embryo quality and pregnancy rates in older patients remain markedly lower than those in younger women. In this study, we investigated whether EZTG improves oocyte quality in a naturally aged mouse model by enhancing antioxidant defenses. Our results demonstrate that EZTG activates the Nrf2/HO-1 signaling pathway, upregulates antioxidant enzymes (SOD, CAT, GPX1), reduces ROS levels, and attenuates oxidative stress-induced cellular damage. Additionally, EZTG restored estrous cyclicity, improved ovarian function, increased follicle counts, and reduced follicular atresia in aged mice. These effects were dose-dependent, with the high-dose group exhibiting the most pronounced improvements.

During follicular development, granulosa cell apoptosis is a key factor contributing to follicular atresia [17]. Granulosa cells play an essential role in supporting oocyte maturation by providing nutrients and paracrine signals; therefore, their dysfunction can significantly impair oocyte quality and ovarian reserve. Given their central role, this study focused on granulosa cells as the primary subject of investigation. It is well established that oxidative stress is a major inducer of apoptosis in both oocytes and granulosa cells. Elevated ROS levels activate mitochondria-mediated apoptotic pathways, leading to granulosa cell death and oocyte atresia [18]. Moreover, oxidative stress-induced apoptosis in granulosa cells has been associated with decreased estradiol production, reduced ovulation rates, and compromised oocyte quality [19]. Disruption of granulosa cell-oocyte communication due to oxidative damage further undermines oocyte nourishment and preovulatory maturation [20].

Studies have also demonstrated that granulosa cell apoptosis is age-related, with a higher apoptotic rate observed in granulosa cells from older IVF-ET patients. Consistent with these findings, our results showed that aged mice exhibited significantly elevated ROS levels in granulosa cells compared to young controls, indicating more severe oxidative damage. Additionally, the pro-apoptotic factors Bax and cleaved Caspase-3 were upregulated at both the mRNA and protein levels, whereas the anti-apoptotic factor Bcl-2 was downregulated. These findings confirm that oxidative stress triggers apoptosis in granulosa cells, accelerating ovarian reserve depletion, impairing follicular development and maturation, and ultimately compromising reproductive function. Notably, EZTG treatment effectively enhanced antioxidant defenses and anti-apoptotic capacity in aged mice, with the therapeutic effects displaying clear dose-dependency.

EZTG comprises several well-known Chinese medicinal herbs, including Lycium barbarum (goji berry), Ligustrum lucidum (glossy privet fruit), and Rehmannia glutinosa (prepared rehmannia root), all of which exhibit strong antioxidant and anti-apoptotic properties. Lycium barbarum is noted for its ability to scavenge hydroxyl and superoxide radicals, enhance the activities of antioxidant enzymes such as SOD and CAT, and reduce malondialdehyde (MDA) levels, thereby exerting potent antioxidative effects. Ligustrum lucidum has been shown to suppress Bax expression while upregulating Bcl-2, thereby inhibiting apoptosis, increasing antral follicle counts, and improving ovarian function in premature ovarian failure (POF) models. Rehmannia glutinosa has been reported to enhance antioxidant enzyme activity and improve immune organ function in aging models, further supporting its strong antioxidative potential.

Mitochondrial dysfunction plays a central role in ovarian aging by disrupting the bidirectional communication between oocytes and granulosa cells and impairing oocyte meiotic competence, ultimately reducing embryonic developmental potential [21]. This dysfunction is characterized by pathological features such as reduced mitochondrial membrane potential (MMP), diminished ATP production, and excessive ROS accumulation [22]. In our study, aged mice exhibited significant mitochondrial damage, including decreased MMP and ATP levels along with elevated ROS levels. Notably, EZTG treatment restored MMP and ATP levels in a dose-dependent manner, demonstrating its capacity to alleviate oxidative damage and preserve oocyte functionality.

A decline in MMP is closely associated with excessive ROS production and oxidative stress [23]. Mitochondrial dysfunction initiates a vicious cycle: ROS accumulation disrupts mitochondrial homeostasis, further exacerbating oxidative stress and eventually triggering apoptosis [24]. The Nrf2 signaling pathway plays a crucial role in interrupting this cascade by upregulating antioxidant enzymes (SOD, CAT, GPX1) to restore redox balance [25]. Activation of Nrf2 prevents ROS-induced collapse of MMP, inhibits the opening of the mitochondrial permeability transition pore, and suppresses cytochrome c release and the activation of downstream apoptotic pathways [26].

The Bcl-2 protein family regulates mitochondrial outer membrane permeability, with pro-apoptotic Bax promoting cytochrome c release, while anti-apoptotic Bcl-2 counteracts this process. Caspase-3, the executioner protease, mediates the terminal steps of apoptosis by degrading cytoskeletal components and DNA. In our study, aged granulosa cells exhibited reduced Bcl-2 expression alongside increased levels of Bax and cleaved Caspase-3. EZTG treatment reversed this pro-apoptotic shift by upregulating Bcl-2 and suppressing Bax and cleaved Caspase-3, thereby protecting granulosa cells by inhibiting mitochondrial-dependent apoptosis.

Nrf2 functions as a key sensor of oxidative stress and a master regulator of cellular redox homeostasis. Under stress conditions, Nrf2 mitigates oxidative damage by upregulating antioxidant enzymes such as HO-1 and SOD [27]. With advancing age, decreased Nrf2 expression in oocytes impairs meiotic competence and normal ovarian function. Previous studies have demonstrated that enhancing Nrf2 activity reduces meiotic defects and delays ovarian aging [28]. Conversely, Nrf2-knockout mice exhibit accelerated ovarian aging, characterized by diminished antioxidant capacity and a reduced follicular pool [7, 29], indicating that Nrf2 deficiency disrupts antioxidant signaling, promotes ROS accumulation, and accelerates ovarian senescence. Together, these findings highlight Nrf2 as a promising therapeutic target for preserving ovarian function.

The critical role of Nrf2 in mediating EZTG’s effects was further corroborated by our in vitro experiments using KGN cells. Nrf2 knockdown completely abolished EZTG-induced antioxidant enzyme upregulation, ROS scavenging, anti-apoptotic effects, and mitochondrial protection, confirming that Nrf2 is indispensable for EZTG’s protective actions. These results not only validate the involvement of the Nrf2/HO-1 pathway but also reinforce the therapeutic potential of targeting Nrf2 in ovarian aging.

EZTG, derived from modified *Erzhi Wan* and *Siwu Tang*, contains multiple bioactive compounds that target oxidative stress pathways. For example, specnuezhenide has been shown to activate the Nrf2 antioxidant system, significantly lowering intracellular ROS and alleviating oxidative stress [30]; oleuropein induces Nrf2/HO-1 signaling, promoting ROS scavenging and inhibiting apoptosis [31]; and chlorogenic acid neutralizes free radicals and suppresses ROS generation, thereby protecting cells from oxidative damage [32]. In our study, EZTG treatment significantly upregulated Nrf2 and HO-1 expression while downregulating Keap1 (a negative regulator of Nrf2) in aged granulosa cells, enhancing the overall antioxidant defense system and correlating with improved ovarian function.

Given that EZTG contains multiple bioactive compounds with pleiotropic effects-such as specnuezhenide and oleuropein, which modulate both Nrf2 and AMPK pathways [33]-its therapeutic benefits may extend beyond isolated Nrf2 activation. This notion is supported by emerging evidence that Nrf2 engages in extensive crosstalk with AMPK and SIRT1 signaling cascades [34]. AMPK can directly phosphorylate Nrf2 or activate it via SIRT1-mediated deacetylation, enhancing its nuclear translocation and transcriptional activity [35]. Conversely, Nrf2 activation feedbacks to regulate mitochondrial biogenesis through PGC-1α, a common downstream effector of AMPK/SIRT1 signaling [36]. This interconnected regulation aligns with the holistic multi-target characteristic of traditional Chinese medicine formulations. Future studies using pathway-specific inhibitors or genetically modified models are warranted to dissect the involvement of AMPK/SIRT1 signaling in EZTG-mediated ovarian protection and to determine whether synergistic effects arise from simultaneous modulation of these pathways.

As these beneficial effects were closely associated with the administered doses, particularly in the high-dose group, it is important to evaluate the clinical relevance and safety of such dosing regimens. Although the dose conversion from animals to humans followed standard guidelines, the equivalent human dose may exceed typical clinical usage. However, traditional Chinese medicine formulations like EZTG are often tailored to individual patients, and higher doses can be used under careful supervision. Notably, no signs of toxicity or adverse effects were observed in the treated animals. Nevertheless, comprehensive toxicological studies-including long-term safety and off-target effects-are essential before clinical application. Future research should aim to define the therapeutic window, maximum tolerated dose, and pharmacokinetic profile of EZTG to ensure its safe and effective translation into clinical practice.

Several limitations of this study should be acknowledged. First, although improvements in ovarian reserve and antioxidant markers were demonstrated, fertility outcomes such as pregnancy and live birth rates in mice were not assessed. Second, while our KGN cell data provide functional evidence for Nrf2 pathway activation, direct assessment of Nrf2 nuclear translocation was not performed, limiting the mechanistic depth. Third, the bioactive compounds responsible for EZTG’s effects remain unidentified, and no positive control (e.g., NAC or CoQ10) was included to benchmark efficacy. Fourth, systematic safety evaluations (e.g., liver/kidney function, histopathology) were not conducted. Future studies should address these limitations to provide a more comprehensive evaluation. Addressing these limitations in future investigations-through fertility trials, mechanistic dissection of Nrf2 signaling, identification of active compounds, and systematic safety evaluations-will be essential to provide a more comprehensive understanding of EZTG’s therapeutic potential and facilitate its clinical translation.

## Conclusion

This study is the first to demonstrate that the EZTG Formula enhances the antioxidant capacity of granulosa cells in aged mice by activating the Nrf2/HO-1 signaling pathway, reducing reactive ROS levels, alleviating oxidative stress-induced damage, improving mitochondrial function, and inhibiting granulosa cell apoptosis, thereby improving oocyte quality. These findings provide experimental evidence supporting the clinical application of the EZTG Formula and offer new insights into personalized treatment strategies for infertility in advanced-age patients. Moreover, the observed dose-dependent antioxidant effects of the EZTG Formula lay the groundwork for optimizing its clinical dosing in future applications.

## Supplementary Information

Supplementary Material 1.

Supplementary Material 2.

Supplementary Material 3.

Supplementary Material 4.

Supplementary Material 5.

Supplementary Material 6.

Supplementary Material 7.

Supplementary Material 8.

Supplementary Material 9.

Supplementary Material 10.

Supplementary Material 11.

Supplementary Material 12.

## Data Availability

Data supporting the findings of this study are available from the corresponding author upon reasonable request.
